# Determinants of Suicide and Accidental or Violent Death in the Australian HIV Observational Database

**DOI:** 10.1371/journal.pone.0089089

**Published:** 2014-02-19

**Authors:** Hamish McManus, Kathy Petoumenos, Teo Franic, Mark D. Kelly, Jo Watson, Catherine C. O’Connor, Mark Jeanes, Jennifer Hoy, David A. Cooper, Matthew G. Law, on behalf of the Australian HIV Observational Database

**Affiliations:** 1 The Kirby Institute, University of New South Wales, Sydney, Australia; 2 Holdsworth House GP, Sydney, Australia; 3 Brisbane Sexual Health and HIV Service, Brisbane, Queensland, Australia; 4 National Association of People with HIV Australia, Sydney, Australia; 5 RPA Sexual Health, Royal Prince Alfred Hospital, Sydney, Australia; 6 Central Clinical School, Sydney University, Sydney, Australia; 7 Department of Medicine, Monash University, Melbourne, Victoria, Australia; University of Nebraska Medical Center, United States of America

## Abstract

**Background:**

Rates of suicide and accidental or violent death remain high in HIV-positive populations despite significantly improved prognosis since the introduction of cART.

**Methods:**

We conducted a nested case-control study of suicide and accidental or violent death in the Australian HIV Observational Database (AHOD) between January 1999 and March 2012. For each case, 2 controls were matched by clinic, age, sex, mode of exposure and HIV-positive date to adjust for potential confounding by these covariates. Risk of suicide and accidental or violent death was estimated using conditional logistic regression.

**Results:**

We included 27 cases (17 suicide and 10 violent/accidental death) and 54 controls. All cases were men who have sex with men (MSM) or MSM/ injecting drug use (IDU) mode of exposure. Increased risk was associated with unemployment (Odds Ratio (OR) 5.86, 95% CI: 1.69–20.37), living alone (OR 3.26, 95% CI: 1.06–10.07), suicidal ideation (OR 6.55, 95% CI: 1.70–25.21), and >2 psychiatric/cognitive risk factors (OR 4.99, 95% CI: 1.17–30.65). CD4 cell count of >500 cells/µL (OR 0.25, 95% CI: 0.07–0.87) and HIV-positive date ≥1990 (1990–1999 (OR 0.31, 95% CI: 0.11–0.89), post-2000 (OR 0.08, 95% CI: 0.01–0.84)) were associated with decreased risk. CD4 cell count ≥500 cells/µL remained a significant predictor of reduced risk (OR 0.15, 95% CI: 0.03–0.70) in a multivariate model adjusted for employment status, accommodation status and HIV-positive date.

**Conclusions:**

After adjustment for psychosocial factors, the immunological status of HIV-positive patients contributed to the risk of suicide and accidental or violent death. The number of psychiatric/cognitive diagnoses contributed to the level of risk but many psychosocial factors were not individually significant. These findings indicate a complex interplay of factors associated with risk of suicide and accidental or violent death.

## Introduction

In the era of effective combination antiretroviral treatment (cART) more than half the causes of death in HIV positive patients are non-AIDS related [1]–[5], the most common being non-AIDS defining cancers, cardiovascular disease and liver disease.

High rates of suicide and accidental or violent death have also been described in HIV infected populations including in those receiving effective cART [6]–[8]. The Data Collection on Adverse Events of Anti-HIV Drugs (D:A:D) cohort reported suicide as the cause of death in 4% of deaths, with a further 2.5% attributed to drug overdose and 1.5% as accident [9]. In CASCADE, suicide was reported in 6.4% of deaths, violence in 3.3%, and 5.7% of deaths were attributed to substance abuse [10].

In the early years of the HIV epidemic, poor prognosis was a key contributing factor to high rates of suicide. Common experiences of HIV positive people such as stigmatisation, discrimination, social isolation, anxiety and depression, as well as frequent substance abuse, were also identified as contributory factors to suicide risk. Yet, despite the significant improvement in prognosis since the introduction of cART the rates of suicide remain high. This was demonstrated in the Swiss HIV Cohort Study where rates of suicide decreased substantially in the cART era compared to the pre cART era but still remained well above that observed in the general population [6].

In the Swiss HIV Cohort Study, the majority (>75%) of patients who committed suicide in the cART era had a diagnosis of mental illness with depression being the most common (>80%). A significant proportion (23%) of patients who died by suicide in the cART era had untreated mental illness. In this cohort, suicide rates were shown to decline with increasing CD4 cell counts. Advanced clinical stage (using the US Centres for Disease Control and Prevention classification system [11]) was significantly associated with suicide risk in both pre- and post-cART eras, after adjustment for other socio-demographic factors and history of psychiatric treatment. However, CD4 cell count and other HIV related factors were not included in this risk factor analysis. In the Concerted Action on SeroConversion to AIDS and Death in Europe (CASCADE) study, latest CD4 cell count was not significantly associated with violent causes of death. As with the Swiss HIV Cohort Study, the CASCADE study did not assess risk factors for suicide which take into account both HIV related factors and general risk factors for suicide in one model.

The extent to which HIV infection is also associated with increased risk of suicide or violent death is not well documented. Specifically the relative contribution of HIV related factors such as immune deficiency, as well as aspects of treatment neuroprotection/ neurotoxicity (e.g. cerebro-spinal fluid (CSF) penetrating/non penetrating ART) compared to accepted psychosocial risk factors has not been described in detail. Recent qualitative models of suicidality in HIV populations in the post cART era also incorporate synergistic effects of HIV related factors and ageing [12].

Most HIV-related studies of suicide focus on suicidality as an endpoint [13]. This obviates difficulties associated with low prevalence of suicide cases, case finding and retrospective data collection. However, there are important differences between determinants of suicidal ideation and suicide. For example, in a study of British private households, Gunnell *et al* found differences in the risk pattern of suicidal thoughts compared to completed suicide according to gender and age [14]. In that study the incidence of suicidal thoughts was seen to be over 200 times greater than the incidence of suicide. In this study we analysed determinants of confirmed cases of suicide as well as of accidental or violent death to develop adjusted models of risk associated with both psychosocial and HIV-related factors.

## Methods

### Study Population

The Australian HIV Observational Database (AHOD) is an observational clinical cohort study of patients with HIV infection seen at 27 clinical sites throughout Australia. AHOD utilises methodology which has been described in detail elsewhere [14]. Briefly, data are transferred electronically to The Kirby Institute, University of New South Wales every 6 months. Core data variables include: sex; date of birth; date of most recent visit; HIV exposure; hepatitis B virus (HBV) surface antigen status; hepatitis C virus (HCV) antibody status; CD4 and CD8 counts; HIV viral load; antiretroviral treatment data; AIDS-defining illnesses; and date and cause of death. Prospective data collection commenced in 1999, with retrospective data provided where available.

Ethics approval for the AHOD study was granted by the University of New South Wales Human Research Ethics Committee, and all other relevant institutional review boards. Written informed consent was obtained from participating individuals. All study procedures were developed in accordance with the revised 1975 Helsinki Declaration. Further specific ethics approval was granted for this particular study by the University of New South Wales Human Research Ethics Committee, and all other relevant institutional review boards.

### Classification of causes of death

The primary endpoint for this study was mortality from suicide and accidental or other violent causes and has been classified as ‘Unnatural’ cause of death in a previous AHOD study [15]. Collection of data on cause of death in AHOD has been described in detail elsewhere [3], [15]. Briefly, AHOD has collected detailed information on cause of death (COD) for all deaths occurring since study inception in 1999: from 2001 until 2002 collected by the study coordinator via direct contact with relevant sites and included study participant deaths occurring prior to that period; thereafter until 2005 using a standardized COD form based on the Data collection on adverse events of anti-HIV Drugs (D:A:D) cohort original COD form [16]; and from 2005 using, the more detailed D:A:D cohort CoDe case report form (CRF) for coding causes of death and adapted from ICD-10 codes (http://www.cphiv.dk/Portals/0/files/CRF2012v2.pdf) [17]. Both the initial COD form and later the CoDe are completed by a clinician at the study site with details of autopsy reports appended where relevant and then forwarded to the Kirby Institute for review by AHOD coordinators. If required, an independent HIV specialist clinician verifies the primary and secondary causes of death and in cases where inadequate information is provided to determine the COD, the study coordinator contacts personnel at the study site for further information. Patients are classified as having an unknown COD if no further information is obtained.

For this study, consistency between recorded cause of death and observed medical records was verified during the site visit by the AHOD study coordinator at the time of study specific data collection.

### Statistical methods

This study is a nested case control study from those sites with confirmed cases of suicide and accidental or violent death for patients consented and recruited to AHOD over the period from study inception in 1999 until 31 March 2012. For each case 2 controls were randomly selected from AHOD patients matched to cases by treating clinic, sex, 10-year age group at the time of case death (“20–29”/”30–39”/”40–49”/”50–59”) and mode of exposure (“men who have sex with men (MSM)”/”MSM and IDU”/” IDU”/” Heterosexual”/”Receipt of blood products”/”Other”) to control for confounder effects. Controls were alive and were HIV-positive at the time of case death. Mode of exposure was not included in analyses as an independent predictor, to avoid possible large discrepancy in empiric distributions of modes of exposure between cases and controls. This would substantially reduce the statistical efficiency of analyses incorporating this covariate.

Specific data on possible risk factors associated with suicide or accidental or violent cause of death was collected using a study Case Record Form (CRF) by a single AHOD coordinator (HM) at site visits. An additional 2 case CRFs for 1 site were completed by site psychologists using medical records and previous study documents not available at the time of site visit by the AHOD coordinator. An additional 1 case CRF and 2 associated control CRFs for 1 other site were completed by the principal investigator for that site following determination by the research ethics committee for that site that the AHOD investigator not be granted direct access to those medical records. All CRF data was uploaded to an MS Access database at the Kirby Institute by electronic form entry.

Specific CRF variables used in this analysis included socio-demographic and lifestyle factors, specifically: Employment in the year prior to case death (dichotomised to “Full time employment (FTE)”/”Not FTE”); Accommodation in the year prior to case death (dichotomised to “Alone”/”Not alone”); Record of prior suicide attempts (“Yes”/”No”); Recorded alcohol consumption in the year prior to case death (“No Record” (NR)/” No consumption”/”Consumption”); Specified alcohol amount consumed in year prior to case death; Recorded smoking prior to case death (“NR”/”Never”/”Currently”/”Previously”); Recorded recreational/ illicit drug use (“NR”/”Never”/ “YES-non IDU”/”Yes-IDU”) and Record of recreational/illicit drug use in the year prior to case death.

CRF variables also included the incidence (ever and in year prior to case death) of recorded mental and cognitive and other risk factors, specifically: suicidal ideation or intent; depression/anxiety; agitation; dysthymia; bipolar disorder; schizophrenia; other mood disorders; anorexia nervosa; chronic pain; epilepsy; and dementia. Data was also collected on recorded psychiatric medications (type, use ever, use in the year prior to case death).

CRF detail on specific psychiatric, cognitive and other risk factors was determined and corroborated where possible by repeated clinician entries in medical records, entries by different clinicians, by specialist referrals and diagnoses including psychiatrist notes and psychologist/counselor notes, and by recorded prescriptions of associated medications/ therapies.

An aggregated count of mental and cognitive risk factors (“0”/”1–2”/”>2”) was also calculated as the count of first incidence of these conditions.

Aggregated quantity of alcohol intake (Alcohol) was determined by ranking reported alcohol consumption quantities for the year prior to case death and classifying as “Low“, ”Moderate”, “High” or “Not stated”. Specifically, “Low” was categorised as up to and including 3 standard drinks/month, “Moderate” was up to and including 2–5 standard drinks/night while “High” was all consumption greater than this. Where there was reported alcohol consumption without specified quantity of alcohol consumption, alcohol intake was assigned the median value (“Moderate”).

Other core HIV related variables routinely collected in AHOD and included in this analysis were CD4 cell count, HIV viral load, AIDS defining illnesses (ADI) (“Yes”/”No”) and antiretroviral treatments (efavirenz, nevirapine, abacavir, stavudine, lamivudine and ritonavir). The duration weighted use of cART with cerebro-spinal fluid penetrative effectiveness scores (CPE) greater than the study median (nerocART) in the year prior to case death was also included in analyses. CPE scores were based on the 2010 ranks published by Letendre [18]. NeurocART status was assigned to regimens with CPE greater than the study median for that year.

Neurocognitive impairment (NCI) was included in analyses based on medical record of dementia, or detail listing memory loss, intellectual impariment, encephalopathy or brain damage, or incidence of HIV encephalopathy/AIDS dementia complex (ADC) or progressive multifocal leukoenephalopathy (PML) recorded in AHOD.

Duration weighted average of CD4 cell counts and HIV viral load values from the year prior to case death as predictors of suicide risk were used in analyses. These variables might more accurately model immunological/virological response than using most recent CD4 cell counts and HIV viral load values. Specifically, constant rates of change in CD4 cell counts and HIV viral load values between test dates were assumed over the interval between consecutive tests and these gradients used to calculate duration weighted average CD4 cell counts and HIV viral load values for year prior to case death.

Determinants of suicide and accidental or violent death were analysed using univariate conditional logistic regression, matching cases to controls and using the variables listed above as independent variables. Robust Huber/White variance estimation was used.

Multivariate analysis was conducted using backward stepwise selection from the set of significant predictors based on univariate analyses but was limited by low numbers of observations. Interaction between covariates was examined using bivariate interaction models of significant predictors in multivariate models. A sensitivity analysis investigating determinants of suicide-only death was conducted using univariate conditional logistic regression as above. Data were analysed using Stata version 12 (Stata Corporation, College Station, Texas, USA).

## Results

### Patient characteristics

A total of 81 patients were included in this analysis – 27 cases and 54 controls. Of cases, 17 (63%) were classified as death by suicide with mode of suicide by hanging (5 deaths) and drug overdose (5 deaths) being the most common (Table 1). A further 10 cases (37%) were classified as violent or accidental death, with death with associated drug overdose (8 deaths) being the most common. All cases were male, with a mean age of 41.6 years compared with 41.1 years for controls. Most deaths occurred prior to 2006 (61.6%) and mode of HIV exposure was either MSM (92.6%) or MSM/IDU (7.4%) (Table 1).

**Table 1:** Characteristics used for case control selection^1^.

	CASE		CONTROL	
	n = 27	*%*	n = 54	*%*
**Suicide death (n = 17)**				
hanging	5	*18.5*		
drug overdose	5	*18.5*		
wrist cutting	1	*3.7*		
gas inhalation	1	*3.7*		
jump from height	1	*3.7*		
not specified	4	*14.8*		
**Violent/accidental death (n = 10)**				
drug overdose (direct or associated)	8	*29.6*		
motor vehicle accident	2	*7.4*		
Gender				
Male	27	*100*	54	*100*
**Age at case death**				
mean (SD)	41.6 (1.54)		41.1 (1.11)	
20–29	1	*3.7*	2	*3.7*
30–39	13	*48.1*	26	*48.1*
40–49	8	*29.6*	16	*29.6*
50–59	5	*18.5*	10	*18.5*
**Year of Death**				
1999–2002	11	*40.7*	–	–
2003–2005	7	*25.9*	–	–
2006–2008	5	*18.5*	–	–
2009–2011	4	*14.8*	–	–
**Mode of Exposure**				
MSM	25	*92.6*	50	*92.6*
MSM/IDU	2	*7.4*	4	*7.4*

1. Controls were also matched to cases by treating clinic and were HIV-positive prior to case death.

Patient characteristics prior to case death are shown in Table 2. Cases generally recorded higher levels of risk factors for suicide or violent death. Of social and demographic risk factors, cases were less likely to be in full time employment (59.3% vs. 35.3%), and more likely to live alone or in an institutional environment (55.6% vs. 25.9%). Of mental health, neurocognitive and other medical risk factors, cases were more likely to have a record of suicidal ideation (55.6% vs. 20.4%) and to have an increased number of recorded mental and cognitive and other clinical risk factors (>2 conditions ever recorded (29.6% vs. 11.1%)). Of HIV specific factors, cases were less likely to have recent CD4 count ≥500 cells/µL (18.5% vs. 46.3%). Cases were more likely to have been HIV-positive prior to 1990 (44.4% vs. 22.2%).

**Table 2:** Patient characteristics prior to case death and risk of suicide and accidental/violent death^1^.

		Case		Control					
		n = 27	*%*	n = 54	*%*	OR	95% CI	p	p^2^
Full time employment^3^	Yes	6	*22.2*	33	*61.1*	1.00	-	-	0.002
	No	16	*59.3*	19	*35.2*	5.86	[1.69,20.37]	0.005	
	Not stated (NS)	5	*18.5*	2	*3.7*	28.54	[3.12,261.23]	0.003	
Accommodation^3^	Not alone	7	*25.9*	23	*42.6*	1.00	-	-	0.021
	Alone/Institution	15	*55.6*	14	*25.9*	3.26	[1.06,10.07]	0.040	
	Not stated	5	*18.5*	17	*31.5*	0.63	[0.20,1.96]	0.425	
Alcohol intake^3^	Moderate or less	13	*48.1*	36	*66.7*	1.00	-	-	0.397
	High	5	*18.5*	6	*11.1*	2.31	[0.46, 11.54]	0.308	
	NS	9	*33.3*	12	*22.2*	1.72	[0.61,4.91]	0.196	
Smoking^3^	Never	11	*40.7*	18	*33.3*	1.00	-	-	0.284
	Prior	2	*7.4*	10	*18.5*	0.33	[0.08,1.42]	0.136	
	Current	12	*44.4*	25	*46.3*	0.76	[0.20,2.87]	0.681	
	Not stated	2	*7.4*	1	*1.9*	3.50	[0.25,49.36]	0.354	
Recreational/Illicit drug use ever	No	4	*14.8*	4	*7.4*				
	Non IDU	10	*37*	11	*20.4*				
	IDU	3	*11.1*	5	*9.3*				
	Not stated	10	*37*	34	*63*				
Recreational/Illicit drug use^3^	No	21	*77.8*	47	*87*	1.00	-	-	
	Yes	6	*22.2*	7	*13*	2.35	[0.64,8.70]	0.200	
Suicide attempts	No	21	*77.8*	48	*88.9*	1.00	-	-	
	Yes	6	*22.2*	6	*11.1*	2.73	[0.70,10.69]	0.149	
Suicidal ideation	No	12	*44.4*	43	*79.6*	1.00	-	-	
	Yes	15	*55.6*	11	*20.4*	6.55	[1.70,25.21]	0.006	
Depression/anxiety	No	9	*33.3*	27	*50.0*	1.00	-	-	
	Yes	18	*66.7*	27	*50.0*	1.87	[0.67,5.25]	0.234	
Neurocognitive impairment	No	24	*88.9*	47	*87.0*	1.00	-	-	
	Yes	3	*11.1*	7	*13.0*	0.84	[0.22,3.26]	0.804	
Chronic pain	No	21	*77.8*	49	*90.7*	1.00	-	-	
	Yes	6	*22.2*	5	*9.3*	2.68	[0.74,9.68]	0.132	
Psych/cognitive risks	≤2	19	*70.4*	48	*88.9*	1.00	-	-	
	>2	8	*29.6*	6	*11.1*	4.99	[1.17,30.65]	0.031	
Recent psych/cognitive risks^3, 4^	≤2	24	*88.9*	50	*92.6*	1.00	-	-	
	>2	3	*11.1*	4	*7.4*	1.62	[0.23,11.30]	0.628	
Psychotropic medications	≤2	17	*63*	42	*77.8*	1.00	-	-	
	>2	10	*37*	12	*22.2*	1.76	[0.61,5.08]	0.297	
Recent psychotropic medications^3^	≤2	14	*51.9*	42	*77.8*	1.00	-	-	
	>2	13	*48.1*	12	*22.2*	2.47	[0.95,6.41]	0.062	
AIDS defining illness	No	25	*92.6*	42	*77.8*	1.00	-	-	
	Yes	2	*7.4*	12	*22.2*	0.29	[0.06,1.34]	0.112	
AIDS dementia complex	No	27	*100*	51	*94.4*	1.00	-	-	
	Yes	0	*0*	3	*5.6*	Does not converge	-	-	
NeurocART^5, 6^	No	16	*59.3*	38	*70.4*	1.00	-	-	
	Yes	11	*40.7*	16	*29.6*	1.74	[0.68,4.48]	0.251	
CD4 cell count (cells/µL)^6^	<500	17	*63.0*	26	*48.1*	1.00	-	-	0.022
	≥500	5	*18.5*	25	*46.3*	0.25	[0.07,0.87]	0.030	
	Missing	5	*18.5*	3	*5.6*	1.68	[0.33, 8.48]	0.533	
HIV viral load (copies/ml)^6^	≤400	11	*40.7*	30	*55.6*	1.00	-	-	0.291
	>400	11	*40.7*	21	*38.9*	1.63	[0.61,4.36]	0.368	
	Missing	5	*18.5*	3	*5.6*	3.91	[0.69,22.2]	0.124	
First HIV positive result	<1990	12	*44.4*	12	*22.2*	1.00	-	-	0.04
	1990-1999	10	*37*	28	*51.9*	0.31	[0.11,0.89]	0.030	
	≥2000	5	*18.5*	14	*25.9*	0.08	[0.01,0.84]	0.035	
Efavirenz	No	20	*74.1*	49	*90.7*	1.00	-	-	
	Yes	7	*25.9*	5	*9.3*	3.78	[0.96,14.87]	0.057	
Nevirapine	No	23	*85.2*	36	*66.7*	1.00	-	-	
	Yes	4	*14.8*	18	*33.3*	0.37	[0.12,1.10]	0.073	
Abacavir	No	25	*92.6*	42	*77.8*	1.00	-	-	
	Yes	2	*7.4*	12	*22.2*	0.27	[0.05,1.38]	0.115	
Stavudine	No	23	*85.2*	46	*85.2*	1.00	-	-	
	Yes	4	*14.8*	8	*14.8*	1.00	[0.24,4.11]	1.000	
Lamivudine	No	17	*63*	28	*51.9*	1.00	-	-	
	Yes	10	*37*	26	*48.1*	0.53	[0.17,1.72]	0.293	
Ritonavir	No	22	*81.5*	40	*74.1*	1.00	-	-	
	Yes	5	*18.5*	14	*25.9*	0.66	[0.20, 2.14]	0.488	

1. Controls matched to cases by treating clinic, sex, age (10 year age category at case death), mode of exposure, HIV-positive at case death. Univariate conditional logistic regression used to determine OR.

2. Overall Wald test for categorical variables.

3. Record in year prior to case death.

4. Count of recorded suicidal ideation, depression/anxiety, dysthymia, mood and personality disorders, anorexia nervosa, schizophrenia, neurocognitive impairment.

5. NeurocART regimens are those with CPE greater study median per calendar year of case death.

6. Duration weighted average in year prior to case death.

### Univariate analysis

Factors significantly associated with increased risk of suicide and accidental or violent death (Table 2) were employment (not in full time employment (p = 0.005), not stated (p = 0.003)), living alone or in an institution (p = 0.021), record of suicidal ideation (p = 0.006) and more than two recorded psychiatric/cognitive risk factors (p = 0.031)). Factors significantly associated with a reduced risk of suicide or accidental or violent death were recent CD4 cell count greater than or equal to 500 cells/µl (p = 0.022) and year HIV-positive after 1990 (1990–1999 (p = 0.03), ≥2000 (p = 0.035).

### Multivariate analysis

To facilitate fitting of the multivariate model given low numbers of observations, certain inclusion variables were dichotomised. A multivariate model was initially fit with recorded recent full time employment (FTE) (“Y”/”N”), recorded recent accommodation status of alone/institution (“Y”/N”), psychiatric/cognitive risk factor count (“≤2”/”>2”), year HIV positive (“<2000”/”≥2000”) and duration weighted average CD4 cell count (“<500”/”≥500”/”Missing”). Record of suicidal ideation was excluded from model fitting because of collinearity associated with prior inclusion of psychiatric/cognitive risk factor count. The variables retained in the final model were average recent CD4 (p = 0.013), recent full time employment (p = 0.021), recent accommodation status of alone/institution (p = 0.005) and year HIV positive (p = 0.004) (Table 3).

**Table 3:** Multivariate conditional logistic regression of determinants of suicide and accidental/violent death^1^.

		Case		Control					
		n = 27	*%*	n = 54	*%*	OR	95% CI	P	P^2^
Full time employment^3^	Yes	6	*22.2*	33	*61.1*	1.00	-	-	
	No/NS	21	*77.8*	21	*38.9*	14.25	(1.49, 136.17)	0.021	
Accommodation (alone/institution)^3^	No/NS	12	*44.4*	40	*74.1*	1.00	-	-	
	Yes	15	*55.6*	14	*25.9*	4.66	(1.59, 13.68)	0.005	
CD4 cell count (cells/µL)^4^	<500	17	*63.0*	25	*46.3*	1.00	-	-	0.013
	≥500	5	*18.5*	24	*44.4*	0.15	(0.03, 0.70)	0.016	
	Missing	5	*18.5*	5	*9.3*	2.40	(0.37, 15.44)	0.358	
First HIV positive result	<2000	22	*81.5*	40	*74.1*	1.00	-	-	
	≥2000	5	*18.5*	14	*25.9*	0.07	(0.01, 0.41)	0.004	

P>chi^2^ = 0.019, Pseudo R^2^ = 0.467.

1. Controls matched to cases by treating clinic, sex, age (10 year age category at case death), mode of exposure, HIV-positive at case death.

2. Overall Wald test for categorical variables.

3. Record in year prior to case death.

4. Duration weighted average in year prior to case death.

### Suicide-only death

Low numbers of endpoints prevented detailed examination of suicide-only death, particularly for non-binary categorical variables with unreported/missing values. Models were otherwise qualitatively similar to those developed for the primary analysis (results not shown). Record of suicidal ideation ever was the only significant predictor of risk of suicide (OR 7.88, 95% CI 1.44-43.06, p = 0.020).

## Discussion

This study found multivariate association between increased risk of suicide and accidental or violent death in HIV-positive patients, psychosocial factors (employment and accommodation status) and recent immunological status while controlling for age, sex, mode of exposure, treating clinic and calendar year of HIV diagnosis.

We found an increased prevalence of observed psychosocial risk factors in cases which reflects well documented prognostic indicators [7], [19]. In particular, employment, accommodation status and suicidal ideation were associated with increased risk. In this study previously identified risk factors were not individually prognostic (alcohol intake, smoking status, recent recreational/illicit drug use, depression/anxiety, chronic pain and NCI). This is attributable to insufficient difference in prevalence between cases and controls for the given statistical power of this analysis. These results are also consistent with a high prevalence of psychosocial risk factors in HIV-positive populations in particular, but also in medically ill populations in general [20]–[23].

The observed significance of increased number of psychiatric/cognitive risk factors in analyses is consistent with the exacerbation of overall risk by cumulative burden of illnesses. Of recorded psychiatric/cognitive risk factors, only record of suicidal ideation was statistically significant, however, number of diagnoses of psychiatric/cognitive risk factors (≤2/>2 risk factors) was prognostic in univariate models. This variable may also more accurately reflect psychiatric/cognitive status by mitigating specific omissions or oversights as logged in and obtained from medical records.

This analysis found a recent CD4 cell count of ≥500 cells/µL to be a significant predictor of reduced risk of death by suicide, violence or accident. This remained significant in multivariate models after adjustment for socio-demographic factors (employment and accommodation status). The association of increased CD4 cell count and reduced risk has been demonstrated by Keiser *et al* [24], although that analysis did not adjust for other risk factors. Our study uses average recent CD4 cell count which reduces the influence of anomalous or non-characteristic measurements compared to single test values.

Models of suicide risk in HIV-positive populations generally incorporate complex causal pathways. In particular, psychosocial risk factors have been shown to predispose patients to non-adherence [25], [26] hence poorer HIV disease control. There are also reciprocal causal effects of immunological status on severity of neuropsychiatric symptoms as demonstrated by Warriner *et al* [27]. In our analysis there was no evidence of strong interaction between covariates, although robustness of multivariate models was limited by statistical power. In these models reduced level of immunological recovery can be seen to add to the overall risk associated with just psychosocial factors and may also indicate comorbidity effects specifically associated with HIV-disease.

We found earlier calendar year of HIV diagnosis to be associated with increased risk. This study only looks at post cART era mortality, when improved prognosis has been associated with some alleviation of psychosocial burden and reduced suicide risk [24]. However, increased all-cause mortality in HIV populations has been shown to be mainly attributable to risk factors identifiable prior to, or at early stages of cART [25]. While our results in part reflect increased experience of suboptimal therapy amongst high risk groups, they also suggest that extended duration of infection increases risk as has been observed in other chronic medical conditions [28].

Posited mechanisms for suicide risk in HIV-positive populations include the extent of neuro-protection and neuro-toxicity of ART although this was not supported by this analysis. In this study the recent use of neurocART was not significantly associated with risk in univariate analyses. Generally, there was no association between risk and neurocognitive impairment although increased observations are required to support these findings. Similarly, no specific antiretrovirals were associated with risk of suicide or death by violence or accident.

In this analysis all observed cases had reported mode of exposure as “MSM” or “MSM/IDU”. This reflects the composition of the AHOD cohort (>75% of exposures were via these modes) and of the wider epidemic in Australia, rather than necessarily the relative risk compared to other modes of exposure. In this study, cases and controls were matched on “MSM” or “MSM/IDU” mode of exposure and analyses therefore exclude confounding effects on risk arising from other modes of exposure in controls.

Generally, for case-control studies estimated relative risks are influenced by any causal association with case matching variables. Age, gender, MSM status and IDU history, which were categories used for case matching, have well documented association with the prevalence of general risk factors such as recent drug use, alcohol consumption, smoking status and psychiatric and cognitive risks. Given relatively low case numbers, this was an efficient way to conduct adjusted analyses of epidemic specific and less strongly associated predictors such as HIV related factors. Our results however, do not reflect risk associated with different categories of case matching variables.

This study has a number of limitations. First, primary analyses were based on suicide and accidental or violent death although correlates of accidental or violent death potentially differ from those of suicide only death. In this study, CoDe forms, which record detail of cause of death, facilitated the use of this endpoint to capture extra cases with likely similar associated risk behaviours. Specifically, eight of ten accidental or violent deaths were by overdose and the associated degree of intentionality could not be determined. While this may represent some loss of specificity, there is well documented association between drug use disorders and completed suicide [29]. Recently Bohnert *et al* have shown increased likelihood of US medical examiners to classify overdose deaths in cases with substance use disorders as indeterminate intent or unintentional compared to other psychiatric disorders and suggested that this might therefore indicate misclassification of suicide deaths for this group [30]. It is also possible that an additional proportion of accidental or violent deaths are in fact true suicide deaths but where official pronouncement of suicide might have been discouraged because of moral and legal implications [31]. Specifically in this analysis, deaths are very likely due to suicide or to have associated suicidal ideation. The increase in statistical power associated with the expanded endpoint has permitted more detailed analysis than possible by using suicide only death and should be considered in further analyses.

Second, there is a low absolute number of observed suicide deaths in AHOD. In this study we examined the expanded endpoint of suicide to include accidental or violent death and used efficient statistical methods including a case control design nested on certain accepted prognostic factors. However, results need to be carefully interpreted and the chance selection of non-associated variables in models cannot entirely be discounted. However, it is of interest that our findings are consistent with other analyses, as described [7], [19], [21]–[24].

Third, there may be site specific differences in comprehensiveness of reporting of certain risk factors. For example, certain treating centres restrict detailing of illicit drug use in patient records because of possible legal implications. Omitted data is not expected to introduce bias into analyses because of the case-control design of the study which matched cases and controls at the same treating centre.

In conclusion, immunological status of HIV-positive patients was found to add to the level of risk of suicide and accidental or violent death after adjustment for psychosocial factors. Number of psychiatric/cognitive diagnoses contributed to the level of risk but many psychosocial factors were not individually significant predictors of risk. These findings indicate a complex interplay of factors associated with risk of suicide and accidental or violent death.
